# Effectiveness of Antibiotic Prophylaxis in Non-emergency Cholecystectomy Using Data from a Population-Based Cohort Study

**DOI:** 10.1007/s00268-017-4018-3

**Published:** 2017-04-25

**Authors:** Ravinder S. Vohra, James Hodson, Sandro Pasquali, Ewen A. Griffiths, on behalf of the CholeS Study Group and West Midlands Research Collaborative

**Affiliations:** 1 Nottingham Oesophago-Gastric Unit, Nottingham University Hospitals NHS Trust, City Hospital Campus, Hucknall Road, Nottingham, NG5 1PB UK; 2 Institute of Translational Medicine (ITM), University Hospitals Birmingham NHS Foundation Trust Queen Elizabeth Hospital Birmingham, Mindelsohn Way, Edgbaston, Birmingham, B15 2G UK; 3 Surgical Oncology Unit, Veneto Institute of Oncology IOV-IRCCS, Padua, Italy; 4 Department of Upper Gastro-Intestinal Surgery, University Hospitals Birmingham NHS Foundation Trust Queen Elizabeth Hospital Birmingham, Mindelsohn Way, Edgbaston, Birmingham, B15 2GW UK; 5 Institute of Cancer and Genomic Sciences, College of Medical and Dental Sciences, University of Birmingham, Birmingham, UK

## Abstract

**Background:**

There is a variation in the administration of antibiotics prophylaxis to reduce the perceived risk of SSI in patients undergoing non-emergency cholecystectomy. The aim of this study was to determine the effectiveness of antibiotic prophylaxis following non-emergency cholecystectomy to prevent 30-day superficial surgical site infections (SSIs) using non-selected, nationally collected, prospective data.

**Methods:**

Data were extracted from the CholeS study, which examined and independently validated the outcomes on consecutive patients following non-emergency cholecystectomy across 166 hospitals in the UK and Ireland. Patients who received antibiotic prophylaxis were exact matched to those who did not on variables associated with antibiotic prophylaxis. The primary outcome of interest was superficial SSI, and secondary outcomes included deep SSI, readmissions, complications and re-interventions within 30 days.

**Results:**

Out of a total of 7327 patients included in the study, 4468 (61%) received antibiotic prophylaxis. These were matched to patients who did not receive antibiotic prophylaxis on a range of demographic and surgical factors, leaving 1269 pairs of patients for analysis. Within this cohort, patients receiving antibiotic prophylaxis had significantly lower rates of superficial SSI (0.7% vs. 2.3%, *p* = 0.001) and all-cause complications (5.8 vs. 8.0%, *p* = 0.031), but similar rates of deep SSI (1.0 vs. 1.4%, *p* = 0.473), readmissions (5.2 vs. 6.2%, *p* = 0.302) and re-interventions (2.6 vs. 3.7%, *p* = 0.093). The number needed to treat to prevent one superficial SSI was 45 (95% confidence interval 24–662).

**Conclusions:**

Antibiotics appear effective at reducing SSI after non-emergency cholecystectomy. However, due to the high number needed to treat it is unclear whether they provide a worthwhile clinical benefit to patients.

**Electronic supplementary material:**

The online version of this article (doi:10.1007/s00268-017-4018-3) contains supplementary material, which is available to authorized users.

## Introduction

There are wide variations in management of patients undergoing non-emergency cholecystectomy. One example is antibiotics prophylaxis administered to reduce the perceived risk of surgical site infections (SSIs) [1, 2]. A recent systematic review of 19 randomised controlled trials considered 5259 participants undergoing cholecystectomy for biliary colic or mild and moderate acute calculous cholecystitis. Antibiotic prophylaxis was administered to 2709 (51.5%) patients, and this failed to reduce the risk of SSI or overall nosocomial infections [3]. Of note, the majority of studies analysed excluded patients perceived at high risk of SSI, e.g. converted operations and when intra-operative cholangiography was performed.

Current guidelines from the USA and the UK do not recommend antibiotic prophylaxis in non-emergency cholecystectomy for low- or moderate-risk groups, due to the low risk of developing SSIs and cost to the health care system [4–7]. Despite this, between 20 and 80% of patients undergoing non-emergency cholecystectomy are administered antibiotic prophylaxis in nationally collected data sets [1, 5]. The rationale provided by some clinicians is a perceived increased risk of SSI as a result of intra-operative contamination with bile, stones or blood and a lack of pragmatic, effectiveness studies.

The Clinical Variation in Practice of Cholecystectomy and Surgical Outcomes Study (CholeS study) was a multicentre prospective, population-based cohort study of variation in practice of cholecystectomy [8]. It examined and independently validated the 30-day outcomes on 8914 consecutive patients following both emergency and non-emergency cholecystectomy across 166 hospitals in the UK and Ireland between 1 March and 1 May 2014. The main results of the CholeS study have been recently published [9–12]. Using this non-selected, validated data of consecutive patients, the aim of this study was to determine the effectiveness of antibiotic prophylaxis following non-emergency cholecystectomy to prevent 30-day SSI.

## Methods

This was a secondary analysis of the CholeS study [8]. The CholeS study collected anonymous observational data and did not require research registration as confirmed by the online National Research Ethics Service (NRES) decision tool (http://www.hra-decisiontools.org.uk/research/) and further supported by written confirmation and advice from the Research and Development Director at University Hospitals Birmingham NHS Foundation Trust, UK.

The study was registered as a ‘clinical audit’ or ‘service evaluation’ at each participating hospital under the supervision of a named senior investigator (consultant surgeon).

### Inclusion and exclusion criteria

The CholeS study-enrolled patients undergoing cholecystectomy for benign gallbladder diseases in acute UK and Irish hospitals participating in this study between 1 March and 1 May 2014 were included. This secondary analysis investigated the group of patients undergoing non-emergency cholecystectomy for benign gallbladder diseases. Patients undergoing non-emergency cholecystectomy as delayed operations (defined as a scheduled cholecystectomy following an emergency admission with gallbladder disease) or elective operations (defined as a planned elective admission for cholecystectomy referred by their family doctor and added to the routine surgical waiting list from the outpatient department with no prior emergency admission with gallbladder disease) were included. Patients undergoing an emergency cholecystectomy were thus excluded. Open, laparoscopic and laparoscopic converted to open surgeries were included. Patients who had a cholecystectomy for known gallbladder cancer or as a part of another surgical procedure, e.g. pancreaticoduodenectomy, bariatric, anti-reflux or transplant operations or an emergency cholecystectomy (defined as a cholecystectomy during an acute admission) were excluded.

### Outcome measures

The primary outcome of interest was superficial SSI within 30 days. Secondary outcomes included a deep SSI, all-cause readmissions, all-cause complications, all re-interventions and post-operative administration of antibiotics within 30 days. These have been defined previously [9]. Briefly, the following definitions were used:

- Superficial SSI: (1) Purulent drainage from the incision; OR (2) At least two of: pain or tenderness; localised swelling; redness; heat; fever; AND. The incision is opened deliberately to manage infection or the clinician diagnoses a surgical site infection; OR (3) Wound organisms AND pus cells from aspirate/swab;
- Deep SSI: (1) A clinical diagnosis of wound infection with dehiscence of any layer below fat/Scarpa’s fascia; (2) A clinical diagnosis of intra-abdominal collection (fever or abdominal pain) with operative or radiological evidence of a collection;
- Re-interventions: a composite outcome of antibiotics, radiological drainage, re-laparoscopy, laparotomy.

### Data quality

To standardise data quality, a quality assurance programme was developed [8]. This included a detailed study protocol, pilot phase and a requirement for a minimum of 95% data completeness at submission. Case ascertainment and data accuracy were further validated by independent investigators at selected hospitals, who checked data correctness from 2077 (23.3%) patients against original medical records. These independent investigators were not involved in the original data collection. Case ascertainment and accuracy of collected data were above 95.2 and 99.2%, respectively.

### Explanatory variables

The main explanatory variable was the antibiotic prophylaxis, defined as antibiotics administered at induction or during the operation. Other pre- and peri-operative characteristics were considered as potential explanatory variables influencing the primary outcomes. A full list including definitions has been published previously [8]. Briefly, patient characteristics included here were: age, sex, American Society of Anaesthesiologists (ASA) fitness grade (1, normal healthy patient; 2, mild systemic disease; >2, severe systemic disease/severe systemic disease that is a constant threat to life/moribund patient who is not expected to survive without the operation), body mass index (BMI in kg/m^2^; <24.9, 25.0–29.9, 30.0–34.9, >35.0), indication [biliary colic, cholecystitis, pancreatitis, common bile duct (CBD) stones and others]. Surgical factors considered were grade of operating surgeon (non-consultant grade; consultant), operative method (laparoscopic, converted to open), operative difficulty (as defined by the Nassar scale of difficulty for cholecystectomy from 1 to 4 [13]) and intra-operative events (bile spilt, stones spilt, bleeding, bowel Injury, common bile duct injury (CBD) injury, cholangiogram, CBD explored). In addition, a composite variable of ‘high-risk’ group for developing an SSI was generated, including patients aged over 60 years, undergoing an intra-operative cholangiogram, CBD exploration, conversion to open, bile spilt, stones spilt, CBD injury or bowel injury during surgery. These factors have been identified in guidelines to represent patients at potential risk of developing a SSI [7]. Low-risk surgery was considered as patients undergoing surgery with none of the above risk factors.

### Statistical analysis

Initially, univariable analyses were performed to assess the relationships between antibiotic prophylaxis use, and a range of patient and surgical factors, as well as patient outcomes. Continuous variables were compared using independent samples *t* tests. Nominal variables were analysed using Chi-square tests, with Kendall’s tau used for ordinal variables, to account of the ordering of the categories.

To adjust for factors found to be associated with antibiotic prophylaxis, multivariable binary logistic regression models were produced for each outcome. Antibiotic prophylaxis was entered into the model as a factor, and all of the other potentially confounding factors were considered for inclusion, with a forward stepwise entry procedure used to select independent predictors of outcome.

The data were also analysed using a matched approach. Patients who received antibiotic prophylaxis were exact matched to those who did not on all of the confounding factors considered, with age treated as categorical. The outcomes were then compared across the resulting pairs of patients using McNemar’s test, and odds ratios (OR) with 95% confidence interval (CI) were produced, in order to compare the results to those of the multivariable analysis for consistency.

Finally, to test whether the effect of antibiotic prophylaxis differed by patient risk, an additional set of multivariable binary logistic regression models were produced. The patient risk group, antibiotic prophylaxis and the interaction term were entered into the model. One model included the whole cohort of patients, so confounding factors were added to the model using a forward stepwise approach, to give adjusted odds ratios. The analysis was also repeated for the subgroup of matched patients, which did not require this adjustment. The significance of the interaction terms was from these models which were interpreted as testing the difference in the effectiveness of antibiotic prophylaxis in the low- and high-risk groups.

All analyses were performed using IBM SPSS 22 (IBM Corp. Armonk, NY). Patients with missing data were excluded on a per-analysis basis, and p < 0.05 was deemed to be indicative of statistical significant throughout.

## Results

### Study cohort

Data were collected on 8914 patients undergoing a cholecystectomy between 1 March 2014 and 1 May 2014. Of these, 7400 (83.0%) patients underwent non-emergency cholecystectomy. Antibiotic prophylaxis data were unavailable in 73 cases, which were excluded, leaving 7327 cases for analysis. General patient demographics are presented in Table 1. Patients were predominantly female (*n* = 5482, 74.8%) and had a mean age of 51.1 years (SD = 16.3), and antibiotic prophylaxis was administered in 4468 (61.0%) cholecystectomies.

**Table 1:** Patient characteristics

		All patients (*n* = 7327)	No antibiotic prophylaxis (*n* = 2859)	Antibiotic prophylaxis (*n* = 4468)	*P* value
Age (years)	Mean (SD)	51.1 (16.3)	49.5 (16.0)	52.1 (16.4)	<0.001
Gender	Female	5482 (74.8%)	2288 (80.0%)	3194 (71.5%)	<0.001
	Male	1845 (25.2%)	571 (20.0%)	1274 (28.5%)	
BMI	<25	1492 (21.2%)	606 (21.7%)	886 (20.8%)	0.217
	25.1–30.0	2506 (35.6%)	1013 (36.2%)	1493 (35.1%)	
	30.1–35.0	1718 (24.4%)	647 (23.1%)	1071 (25.2%)	
	>35.0	1333 (18.9%)	529 (18.9%)	804 (18.9%)	
ASA grade	1	2844 (39.1%)	1259 (44.3%)	1585 (35.7%)	<0.001
	2	3741 (51.4%)	1370 (48.3%)	2371 (53.4%)	
	>2	692 (9.5%)	210 (7.4%)	482 (10.9%)	
Indication	Colic	4326 (59.1%)	1900 (66.5%)	2426 (54.3%)	<0.001
	Cholecystitis	1753 (23.9%)	529 (18.5%)	1224 (27.4%)	
	Pancreatitis	579 (7.9%)	192 (6.7%)	387 (8.7%)	
	CBD stone	483 (6.6%)	148 (5.2%)	335 (7.5%)	
	Other	182 (2.5%)	87 (3.0%)	95 (2.1%)	
Admission type	Elective	4095 (55.9%)	1800 (63.0%)	2295 (51.4%)	<0.001
	Delayed	3232 (44.1%)	1059 (37.0%)	2173 (48.6%)	
Grade of operating surgeon	Non-consultant	1516 (20.7%)	659 (23.1%)	857 (19.2%)	<0.001
	Consultant	5808 (79.3%)	2198 (76.9%)	3610 (80.8%)	
Operative method	Laparoscopic	7109 (97.0%)	2834 (99.1%)	4275 (95.7%)	<0.001
	Converted to open	218 (3.0%)	25 (0.9%)	193 (4.3%)	
Nassar operative	1	3149 (43.2%)	1550 (54.7%)	1599 (36.0%)	<0.001
Difficulty	2	2248 (30.9%)	854 (30.1%)	1394 (31.4%)	
	3	1365 (18.7%)	369 (13.0%)	996 (22.4%)	
	4	519 (7.1%)	62 (2.2%)	457 (10.3%)	
Bile spilt	Yes	1866 (25.6%)	342 (12.1%)	1524 (34.2%)	<0.001
	No	5416 (74.4%)	2483 (87.9%)	2933 (65.8%)	
Stones spilt	Yes	616 (8.5%)	85 (3.0%)	531 (11.9%)	<0.001
	No	6656 (91.5%)	2739 (97.0%)	3917 (88.1%)	
Bleeding	Yes	548 (7.5%)	140 (5.0%)	408 (9.2%)	<0.001
	No	6724 (92.5%)	2686 (95.0%)	4038 (90.8%)	
Bowel injury	Yes	39 (0.5%)	7 (0.2%)	32 (0.7%)	<0.001
	No	7229 (99.5%)	2816 (99.8%)	4413 (99.3%)	
CBD injury	Yes	18 (0.2%)	0 (0.0%)	18 (0.4%)	<0.001
	No	7192 (99.8%)	2771 (100.0%)	4421 (99.6%)	
IOC	Yes	737 (10.1%)	209 (7.4%)	528 (11.9%)	<0.001
	No	6541 (89.9%)	2617 (92.6%)	3924 (88.1%)	
CBD explored	Yes	162 (2.2%)	30 (1.1%)	132 (3.0%)	<0.001
	No	7110 (97.8%)	2793 (98.9%)	4317 (97.0%)	

*p* Values between antibiotics prophylaxis and no antibiotic prophylaxis groups are from Chi-square tests for nominal variables, Kendall’s tau for ordinal variables and *t* tests for continuous variables; *SD* standard deviation, *BMI* body mass index, *ASA* American Society of Anaesthesiologists; *CBD* common bile duct, *IOC* intra-operative cholangiogram

### Antibiotic prophylaxis use

Patients receiving antibiotic prophylaxis were significantly older and more likely to be male, to have a delayed admission, be operated on by a consultant, have surgeries converted to open and to have greater ASA and operative difficulty (Table 1). Antibiotic prophylaxis also differed significantly by primary indication, being most commonly used in patients with previous cholecystitis and least common in those with biliary colic or ‘other’ indications (Table 1). All the intra-operative events (bile spilt, stones spilt, bleeding, bowel Injury, CBD injury, cholangiogram, CBD explored) were associated with significantly increased likelihood of antibiotic prophylaxis usage (Table 1).

### Outcomes at 30 days

The numbers of patients at 30 days with a superficial SSI was 147 (2.0%), deep SSI 140 (1.9%), all-cause complications 714 (9.7%), post-operative antibiotics use 389 (5.3%) and re-interventions 416 (5.7%). Patients receiving antibiotic prophylaxis were observed to have significantly higher rates of all-cause complications, re-interventions and the requirement for post-operative antibiotics (Table 2). However, this analysis did not account for the significant selection bias in the antibiotic prophylaxis that was previously identified.

**Table 2:** Univariable analysis of outcomes

	No antibiotic prophylaxis (*n* = 2859)	Antibiotic prophylaxis (*n* = 4468)	*P* value
Superficial SSI	61 (2.1%)	86 (1.9%)	0.550
Deep SSI	38 (1.3%)	102 (2.3%)	0.004
All-cause readmissions	182 (6.4%)	300 (6.7%)	0.595
All-cause complications	236 (8.3%)	478 (10.7%)	<0.001
All re-interventions^a^	126 (4.4%)	290 (6.5%)	<0.001
Post-operative antibiotics	118 (4.1%)	271 (6.1%)	<0.001

*p* Values from Chi-square tests

^a^Composite outcome combining post-operative antibiotics, radiological drainage, re-laparoscopy and laparotomy

*SSI* surgical site infection

In order to account for the effect of selection bias, a set of multivariable analyses were performed to test the impact of antibiotic prophylaxis, after accounting for all of pre- and peri-operative factors in Table 1. The adjusted odds ratios from these analyses are reported in Table 3, and full details of the models are reported in Supplementary Tables 1–6. This analysis found superficial SSI to be significantly less likely when antibiotic prophylaxis was administered (OR 0.54, 95% CI 0.37–0.78, *p* = 0.001). No significant association was detected between antibiotic prophylaxis and all-cause readmission, complications, deep SSI or re-interventions.

**Table 3:** Multivariable analyses of outcomes

	Adjusted odds ratio (95% CI)	*P* value
Superficial SSI	0.54 (0.37–0.78)	0.001
Deep SSI	1.12 (0.73–1.71)	0.616
All-cause readmissions	0.87 (0.71–1.07)	0.188
All-cause complications	0.92 (0.77–1.11)	0.395
All re-interventions^a^	0.92 (0.72–1.17)	0.479
Post-operative antibiotics	0.88 (0.69–1.13)	0.328

Odds ratios and *p* values from multivariable binary logistic regression models

^a^Composite outcome combining post-operative antibiotics, radiological drainage, re-laparoscopy and laparotomy

*SSI* surgical site infection

### Matched study

**Fig. 1:**
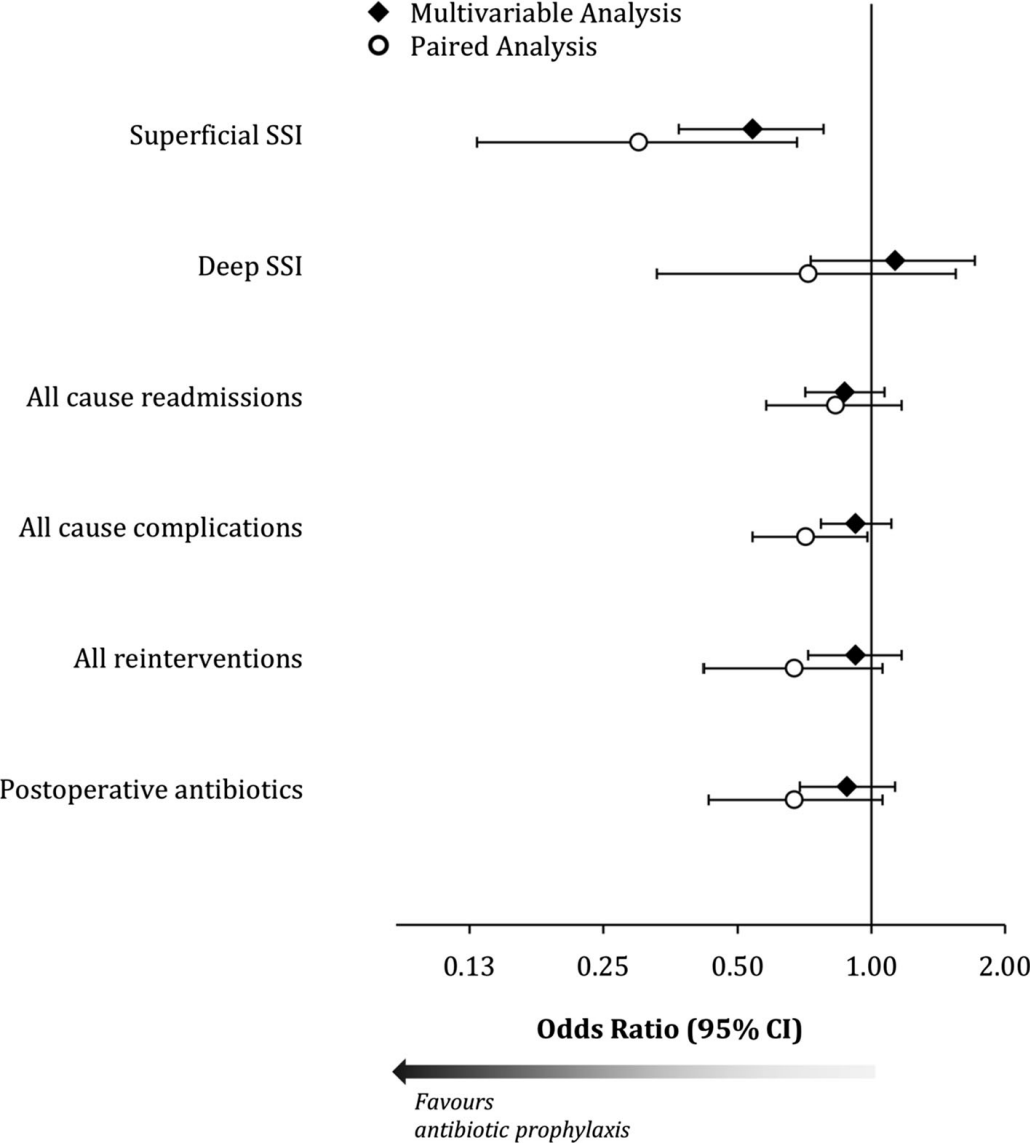
Relationship between antibiotic prophylaxis and outcomes in the multivariable and paired analyses

A paired matched approach was also used to further understand the effect of antibiotic prophylaxis. A total of 1269 pairs of patients could be successfully matched on all of the factors in Table 1 and were included in the analysis. Supplementary Table 7 shows a comparison of patients included in the paired analysis to the remainder of the cohort. The rates of the outcomes being considered were then compared between these two groups (Table 4). Patients receiving antibiotic prophylaxis again had significantly lower rates of superficial SSI (*p* = 0.001), with an odds ratio similar to that from the multivariable analysis (OR 0.30, 95% CI 0.13–0.68, *p* = 0.001). Of patients treated with antibiotic prophylaxis, 0.7% developed SSI, compared to 2.3% in the non-prophylaxis group, giving a number needed to treat (NNT) to prevent one SSI of 63 (95% CI 44–176). The paired analysis also found antibiotic prophylaxis to be associated with a significantly reduced rate of all-cause complications, with an odds ratio of 0.71 (95% CI 0.54–0.98, *p* = 0.031), giving a NNT of 45 (95% CI 24–662). The results of the two statistical approaches are reported graphically in Fig. 1.

**Table 4:** Matched analysis

	No antibiotic prophylaxis (*n* = 1269)	Antibiotic prophylaxis (*n* = 1269)	Odds ratio (95% CI)	*P* value
Superficial SSI	29 (2.3%)	9 (0.7%)	0.30 (0.13–0.68)	0.001
Deep SSI	18 (1.4%)	12 (1.0%)	0.72 (0.33–1.55)	0.473
All-cause readmissions	79 (6.2%)	66 (5.2%)	0.83 (0.58–1.17)	0.302
All-cause complications	102 (8.0%)	74 (5.8%)	0.71 (0.54–0.98)	0.031
All re-interventions^a^	49 (3.9%)	33 (2.6%)	0.67 (0.42–1.06)	0.093
Post-operative antibiotics	47 (3.7%)	32 (2.5%)	0.67 (0.43–1.06)	0.110

*p* values from McNemar’s test

^a^Composite outcome combining post-operative antibiotics, radiological drainage, re-laparoscopy and laparotomy

*SSI* surgical site infection

**Fig. 2:**
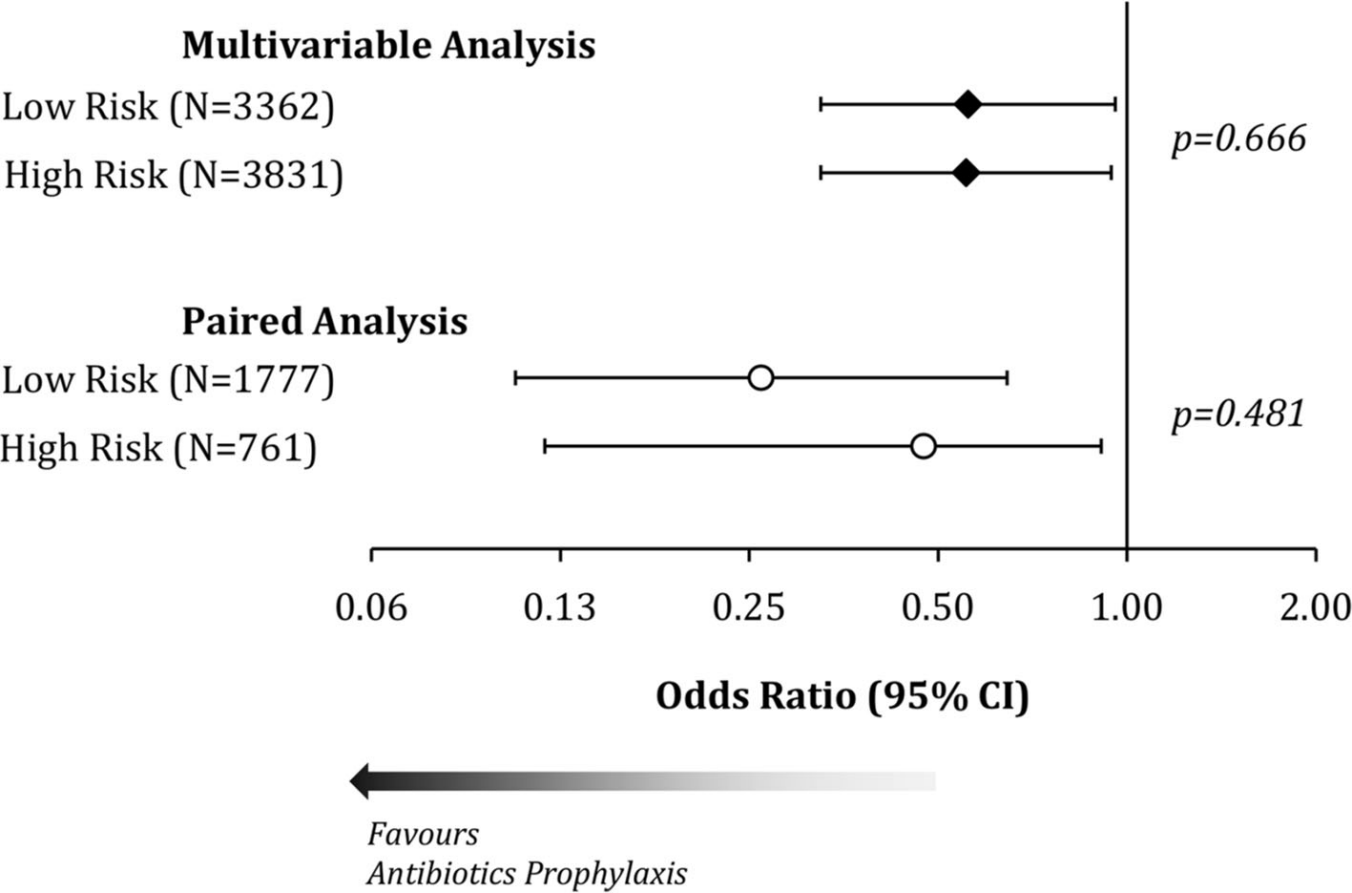
Interaction between antibiotic prophylaxis and patient risk of superficial surgical site infection

An additional analysis was performed to test whether the effectiveness antibiotic prophylaxis differed by low-risk (46.7%) or high-risk (53.3%) patients for a SSI (Fig. 2). The effectiveness of antibiotic prophylaxis to reduce superficial SSI did not differ significantly between the groups in either the multivariable (low risk: OR 0.56, 95% CI 0.33–0.96 vs. high risk: 0.55, 0.33–0.94) or matched (0.26, 0.11–0.64 vs. 0.47, 0.12–0.91) analyses, with *p* = 0.666 and 0.481, respectively.

## Discussion

This study used validated, non-selected population-level data collected as part of the CholeS study [9–12]. In this cohort, 61% of patients undergoing non-emergency cholecystectomy were administered antibiotic prophylaxis. When factors accounting for antibiotic prophylaxis use were adjusted for, antibiotic prophylaxis appeared to reduce superficial SSI at 30 days. This effect was still seen when patients were matched 1-to-1 based on factors related to antibiotic prophylaxis use and did not differ significantly between low- and high-risk patients. There was no evidence antibiotic prophylaxis significantly reduced the rates of deep SSIs, readmissions, post-operative antibiotic use or re-interventions at 30 days.

### Evidence from randomised studies and meta-analyses

The results of this study differ from the current body of evidence of antibiotic prophylaxis in non-emergency cholecystectomy. The majority of randomised studies have showed a small, but non-statistically significant decrease in infective complications when antibiotic prophylaxis is administered. These studies also failed to show a difference in overall SSI rates [3]. The only positive randomised trial showed a significant 2% reduction in SSI with antibiotic prophylaxis using a superiority design [14]. A further non-inferiority randomised trial, which included patients at moderate to high risk of SSI, demonstrated no difference in the antibiotic prophylaxis group using a non-inferiority margin of 11% for the overall infection rate [15]. Taken together with the findings from this study, it does suggest antibiotic prophylaxis reduces certain infective complications.

Two recent meta-analyses have been published comparing the effectiveness of antibiotic prophylaxis in non-emergency cholecystectomy. SSIs were detected in 2.4% of patients given antibiotic prophylaxis and 3.2% who were not [3]. This was however not statistically significant. Another meta-analysis was conducted on 21 trials and 5207 patients who underwent non-emergency cholecystectomy [16]. This found antibiotic usage to significantly reduce the rate of SSI, from 4.0 to 2.6%. However, this 1.4% absolute risk reduction means 70 patients would be needed to be given antibiotic prophylaxis to prevent 1 SSI.

Population-based series have also been analysed. The Swedish Register of Gallstone Surgery and ERCP (GallRiks) study analysed 10,927 patients and found a paradoxical increase in infective complications in the patients given prophylactic antibiotics. This effect was diminished when the results were adjusted for confounding factors, such as age, indication, conversion to open surgery, operative time and gallbladder perforation [1]. The authors concluded that prophylactic antibiotics were unnecessary during non-emergency cholecystectomy, and this has become a quality metric in Sweden.

The results derived from the CholeS study again show an increase in complications in non-adjusted outcomes. Outcomes were then adjusted for patient and surgical factors that influence the administration of antibiotic prophylaxis. In addition, a 1-to-1 paired analysis was performed, where patients were identically matched such that the only difference between the groups was the administration of antibiotic prophylaxis. Antibiotic prophylaxis reduced the rate of superficial SSI from 2.3 to 0.7%, an effect size that was consistent with the analysis that used multivariable adjustment on the whole cohort. This produced a NNT of 63 and 45 in the multivariable adjusted and matched analyses, respectively. This may reflect the inclusion of higher risk and unselected patients in the CholeS study compared to the randomised studies. Even in this analysis, the effectiveness of prophylactic antibiotics remains small.

### Interpretation of our results

One explanation for the differences between our results and current evidence is the lack of studies assessing the effect of antibiotic prophylaxis in high-risk groups. The most recent guidelines from both the UK and the USA suggest antibiotic prophylaxis, but state that it should be considered in groups at high risk of SSI, such as having an intra-operative cholangiogram, bile spillage or conversion to open surgery [7]. However, when the CholeS cohort was divided into high- (53.3%) and low-(46.7%) risk groups, antibiotic prophylaxis was found to be similarly effective at reducing the incidence of superficial SSI, regardless of patient risk.

### Antibiotics’ side effects

Benign gallbladder disease is a major global health burden with an estimated 115 patients for every 100,000 of the world’s population undergoing a cholecystectomy for benign gallbladder disease every year [17]. There is a current global campaign to improve awareness of antibiotic stewardship. One strategy is to reduce inappropriate antibiotic administration, which in part will help address the issue of emerging resistance. In addition, complications of antibiotic use include anaphylaxis, rash and nosocomial infections (e.g. Clostridium difficile), and the additional cost must also be considered. The current studies investigating antibiotic prophylaxis have not been powered to investigate antibiotic-related adverse events or cost effectiveness. Available data is limited and inconclusive on the real burden of adverse reactions [3, 14]. One large study of the use of antibiotic prophylaxis estimated an incidence of Clostridium difficile of up to 1.7% depending upon type of antibiotic, leading to a number needed to harm 1 in 91 [18]. The risks and costs of antibiotic prophylaxis must be balanced against the cost of an SSI, which incurs a cost of up to £3500 in the UK [19]. This has led some to suggest antibiotic prophylaxis should be simply administered during all types of surgeries, even when there is a low risk of SSI [20].

### Study limitations

There are limitations to this study. The data represents a two-month ‘snap-shot’ of practice and may not have fully captured all complications requiring a family doctor attendance. However, it is likely that the proportion of these events is similar in both groups.

In addition, this study prospectively collected and independently validated data obtained utilising trainee-led networks in the UK and Ireland. This methodology is powerful and accurate when studying surgical outcomes [21, 22].

Data were not collected on antibiotic type, as this was left to the discretion of the operating surgeon and guided by local hospital policy. Also, it is unknown whether antibiotics were given pre-operatively, for instance in a difficult case, or intra-operatively, for instance when bile or stones are spilled. In the former case, the use of antibiotics is confounded by neither patients nor disease characteristics, while in the latter the administration of antibiotics is causative. This makes it difficult to interpret the association between antibiotics and infections, and suggests that further research is needed in high-risk patients.

## Conclusions

Despite the current evidence, 61% of the patients in the CholeS data set were administered antibiotic prophylaxis, which is consistent with other population data sets. When factors accounting for antibiotic prophylaxis use were accounted for, either using multivariable adjustment or matched analyses, antibiotic prophylaxis appeared to reduce superficial SSI at 30 days. There was no evidence antibiotic prophylaxis significantly reduced the rates of deep SSIs, readmissions, post-operative antibiotic use or re-interventions at 30 days. The results of this study suggest the need for a high-quality, pragmatic, randomised controlled trial looking at the potential benefit of antibiotic prophylaxis, particularly in high-risk patients.

## Electronic supplementary material

Below is the link to the electronic supplementary material.

Supplementary material 1 (DOCX 33 kb)

Supplementary material 2 (DOCX 33 kb)
