# Supplementary material for: Effectiveness of Antibiotic Prophylaxis in Non-emergency Cholecystectomy Using Data from a Population-Based Cohort Study
Source: World J Surg. 2017 Apr 25;41(9):2231–9. doi: 10.1007/s00268-017-4018-3 (PMC5544819; doi:10.1007/s00268-017-4018-3)
Supplement: Supplementary file 2 — Supplementary material 2 (DOCX 33 kb) [file 268_2017_4018_MOESM2_ESM.docx]

***Supplementary Tables 1-7***

*Results from multivariable binary logistic regression models. The use of antibiotic prophylaxis variable was forced into each model, with the other factors entered using a forwards stepwise approach. All factors in Table 1a and 1b were considered for inclusion.*

*Odds ratios (ORs) for dichotomous factors are for the stated category, using the alternative category as a reference. For factors with more than two categories, the first was used as a reference. Age was categorised based on rounding the quartiles to meaningful values, as there was evidence of poor fit when treated as continuous.*

**Significant at p<0.05*

***Supplementary Table 1 –Multivariable analysis of superficial SSI***

| **Readmissions** | **OR (95% CI)** | **p-Value** |
| --- | --- | --- |
| Antibiotic Prophylaxis (Yes) | 0.54 (0.37 - 0.78) | 0.001* |
| BMI |  | <0.001* |
| *<25* | - | - |
| *25-30* | 1.86 (1.03 - 3.37) | 0.039* |
| *31-35* | 1.82 (0.97 - 3.42) | 0.062 |
| *>35* | 3.35 (1.83 - 6.11) | <0.001* |
| Operative Method (Converted to Open) | 5.28 (2.89 - 9.63) | <0.001* |
| Operative Difficulty |  | <0.001* |
| *1* | - | - |
| *2* | 1.95 (1.24 - 3.07) | 0.004* |
| *3* | 1.60 (0.92 - 2.77) | 0.094 |
| *4* | 3.91 (2.03 - 7.53) | <0.001* |
| Primary Indication |  | 0.024* |
| *Colic* | - | - |
| *CBD Stone* | 1.72 (0.99 - 2.98) | 0.054 |
| *Cholecystitis* | 0.69 (0.43 - 1.10) | 0.119 |
| *Pancreatitis* | 1.43 (0.79 - 2.58) | 0.239 |
| *Other* | 0.42 (0.06 - 3.06) | 0.391 |

***Supplementary Table 2 –Multivariable analysis of deep SSI***

| **Readmissions** | **OR (95% CI)** | **p-Value** |
| --- | --- | --- |
| Antibiotic Prophylaxis (Yes) | 1.12 (0.73 - 1.71) | 0.616 |
| ASA |  | 0.041* |
| *1* | - | - |
| *2* | 1.56 (1.00 - 2.42) | 0.050 |
| *>2* | 2.07 (1.15 - 3.72) | 0.015* |
| BMI |  | 0.012* |
| *<25* | - | - |
| *25-30* | 0.79 (0.51 - 1.23) | 0.295 |
| *31-35* | 0.46 (0.27 - 0.79) | 0.005* |
| *>35* | 0.49 (0.27 - 0.86) | 0.014* |
| Operative Method (Converted to Open) | 3.10 (1.71 - 5.63) | <0.001* |
| Operative Difficulty |  | 0.013* |
| *1* | - | - |
| *2* | 1.27 (0.75 - 2.15) | 0.367 |
| *3* | 2.13 (1.27 - 3.58) | 0.004* |
| *4* | 2.34 (1.22 - 4.49) | 0.010* |
| Bleeding (Yes) | 2.05 (1.29 - 3.26) | 0.002* |
| Bowel Injury (Yes) | 3.45 (1.23 - 9.67) | 0.018* |
| Bile Spilt (Yes) | 1.62 (1.10 - 2.38) | 0.014* |

***Supplementary Table 3 –Multivariable analysis of all cause readmission***

| **Readmissions** | **OR (95% CI)** | **p-Value** |
| --- | --- | --- |
| Antibiotic Prophylaxis (Yes) | 0.87 (0.71 - 1.07) | 0.188 |
| Age |  | 0.001* |
| *<40* | - | - |
| *40-54* | 0.70 (0.54 - 0.90) | 0.006* |
| *55-64* | 0.64 (0.47 - 0.86) | 0.003* |
| *65+* | 0.58 (0.43 - 0.78) | <0.001* |
| ASA |  | 0.025* |
| *1* | - | - |
| *2* | 1.13 (0.90 - 1.42) | 0.280 |
| *>2* | 1.61 (1.14 - 2.27) | 0.007* |
| Admission Type (Elective) | 0.69 (0.57 - 0.84) | <0.001* |
| Operative Method (Converted to Open) | 1.63 (1.04 - 2.57) | 0.034* |
| Bile Split (Yes) | 1.32 (1.06 - 1.65) | 0.012* |
| Bleeding (Yes) | 1.43 (1.04 - 1.97) | 0.029* |
| CBD Explored (Yes) | 2.07 (1.28 - 3.37) | 0.003* |

***Supplementary Table 4 –Multivariable analysis of all cause complications***

| **Readmissions** | **OR (95% CI)** | **p-Value** |
| --- | --- | --- |
| Antibiotic Prophylaxis (Yes) | 0.92 (0.77 - 1.11) | 0.395 |
| Age |  | 0.035* |
| *<40* | - | - |
| *40-54* | 0.75 (0.59 - 0.95) | 0.016* |
| *55-64* | 0.82 (0.63 - 1.06) | 0.133 |
| *65+* | 0.98 (0.77 - 1.26) | 0.893 |
| ASA |  | <0.001* |
| *1* | - | - |
| *2* | 1.14 (0.93 - 1.39) | 0.207 |
| *>2* | 1.72 (1.30 - 2.29) | <0.001* |
| Admission Type (Elective) | 0.73 (0.61 - 0.86) | <0.001* |
| Operative Method (Converted to Open) | 3.07 (2.15 - 4.38) | <0.001* |
| Operative Difficulty |  | <0.001* |
| *1* | - | - |
| *2* | 1.36 (1.10 - 1.67) | 0.004* |
| *3* | 1.23 (0.96 - 1.57) | 0.101 |
| *4* | 1.96 (1.42 - 2.71) | <0.001* |
| CBD Explored (Yes) | 1.79 (1.17 - 2.74) | 0.007* |
| Bile Spilt (Yes) | 1.21 (1.00 - 1.47) | 0.046* |
| Bleeding (Yes) | 1.38 (1.06 - 1.80) | 0.019* |

***Supplementary Table 5 –Multivariable analysis of all reinterventions***

| **Readmissions** | **OR (95% CI)** | **p-Value** |
| --- | --- | --- |
| Antibiotic Prophylaxis (Yes) | 0.92 (0.72 - 1.17) | 0.479 |
| ASA |  | 0.001* |
| *1* | - | - |
| *2* | 1.29 (1.00 - 1.66) | 0.051 |
| *>2* | 1.88 (1.33 - 2.64) | <0.001* |
| Operative Method (Converted to Open) | 2.98 (2.00 - 4.43) | <0.001* |
| Operative Difficulty |  | <0.001* |
| *1* | - | - |
| *2* | 1.36 (1.02 - 1.81) | 0.038* |
| *3* | 1.53 (1.11 - 2.10) | 0.009* |
| *4* | 2.65 (1.77 - 3.96) | <0.001* |
| Bleeding (Yes) | 1.74 (1.28 - 2.36) | <0.001* |
| CBD Injury (Yes) | 6.16 (2.02 - 18.79) | 0.001* |
| Stones Spilt (Yes) | 1.42 (1.04 - 1.93) | 0.028* |
| Primary Indication |  | <0.001* |
| *Colic* | - | - |
| *CBD Stone* | 1.56 (1.07 - 2.27) | 0.021* |
| *Cholecystitis* | 1.27 (0.98 - 1.66) | 0.075 |
| *Pancreatitis* | 2.05 (1.46 - 2.89) | <0.001* |
| *Other* | 0.50 (0.16 - 1.59) | 0.238 |

***Supplementary Table 6 –Multivariable analysis of postoperative antibiotics***

| **Readmissions** | **OR (95% CI)** | **p-Value** |
| --- | --- | --- |
| Antibiotic Prophylaxis (Yes) | 0.88 (0.69 - 1.13) | 0.328 |
| ASA |  | 0.002* |
| *1* | - | - |
| *2* | 1.34 (1.03 - 1.74) | 0.029* |
| *>2* | 1.90 (1.34 - 2.71) | <0.001* |
| Admission Type (Elective) | 0.73 (0.55 - 0.96) | 0.026* |
| Operative Method (Converted to Open) | 3.15 (2.10 - 4.71) | <0.001* |
| Primary Indication |  | 0.012* |
| *Colic* | - | - |
| *CBD Stone* | 1.32 (0.86 - 2.01) | 0.206 |
| *Cholecystitis* | 1.10 (0.81 - 1.51) | 0.539 |
| *Pancreatitis* | 1.84 (1.24 - 2.73) | 0.002* |
| *Other* | 0.35 (0.09 - 1.45) | 0.149 |
| Operative Difficulty |  | <0.001* |
| *1* | - | - |
| *2* | 1.30 (0.97 - 1.75) | 0.082 |
| *3* | 1.53 (1.11 - 2.13) | 0.010* |
| *4* | 2.52 (1.67 - 3.82) | <0.001* |
| Stones Spilt (Yes) | 1.40 (1.01 - 1.92) | 0.042* |
| Bleeding (Yes) | 1.58 (1.14 - 2.17) | 0.005* |
| CBD Injury (Yes) | 4.18 (1.27 - 13.75) | 0.019* |
| Intraoperative Cholangiogram (Yes) | 0.58 (0.39 - 0.88) | 0.010* |
| CBD Explored (Yes) | 1.92 (1.02 - 3.61) | 0.043* |

***Supplementary Table 7 – Comparison of patients included in the paired analysis to the remainder of the cohort***

|  | **Included in**  **Paired Analysis** | **Not Included in Paired Analysis** | | | |
| --- | --- | --- | --- | --- | --- |
|  |  | ***Antibiotic Prophylaxis*** | | | |
|  |  | *No* | *p-Value* | *Yes* | *p-Value* |
| Age*^#^* | 47.0 (0.3) | 51.4 (0.4) | <0.001* | 54.2 (0.3) | <0.001* |
| Gender (Male) | 336 (13.2%) | 403 (25.3%) | <0.001* | 1106 (34.6%) | <0.001* |
| ASA |  |  | <0.001* |  | <0.001* |
| *1* | 1188 (46.8%) | 665 (42.4%) |  | 991 (31.3%) |  |
| *2* | 1256 (49.5%) | 742 (47.3%) |  | 1743 (55.0%) |  |
| *>2* | 94 (3.7%) | 163 (10.4%) |  | 435 (13.7%) |  |
| BMI |  |  | 0.005* |  | <0.001* |
| *<25* | 568 (22.4%) | 322 (21.1%) |  | 602 (20.2%) |  |
| *25-30* | 978 (38.5%) | 524 (34.3%) |  | 1004 (33.6%) |  |
| *31-35* | 548 (21.6%) | 373 (24.4%) |  | 797 (26.7%) |  |
| *>35* | 444 (17.5%) | 307 (20.1%) |  | 582 (19.5%) |  |
| Primary Indication |  |  | <0.001* |  | <0.001* |
| *Colic* | 2000 (78.8%) | 900 (56.7%) |  | 1426 (44.6%) |  |
| *CBD stone* | 44 (1.7%) | 126 (7.9%) |  | 313 (9.8%) |  |
| *Cholecystitis* | 362 (14.3%) | 348 (21.9%) |  | 1043 (32.6%) |  |
| *Pancreatitis* | 98 (3.9%) | 143 (9.0%) |  | 338 (10.6%) |  |
| *Other* | 34 (1.3%) | 70 (4.4%) |  | 78 (2.4%) |  |
| Elective Admissions | 1780 (70.1%) | 910 (57.2%) | <0.001* | 1405 (43.9%) | <0.001* |
| Consultant Surgeon | 2118 (83.5%) | 1139 (71.7%) | <0.001* | 2551 (79.8%) | <0.001* |
| Converted to Open | 4 (0.2%) | 23 (1.4%) | <0.001* | 191 (6.0%) | <0.001* |
| Operative Difficulty |  |  | <0.001* |  | <0.001* |
| *1* | 1476 (58.2%) | 812 (51.9%) |  | 861 (27.1%) |  |
| *2* | 808 (31.8%) | 450 (28.7%) |  | 990 (31.2%) |  |
| *3* | 236 (9.3%) | 251 (16.0%) |  | 878 (27.6%) |  |
| *4* | 18 (0.7%) | 53 (3.4%) |  | 448 (14.1%) |  |
| Bile Spilt | 222 (8.7%) | 231 (14.8%) | <0.001* | 1413 (44.3%) | <0.001* |
| Stones Spilt | 20 (0.8%) | 75 (4.8%) | <0.001* | 521 (16.4%) | <0.001* |
| Bleeding | 32 (1.3%) | 124 (8.0%) | <0.001* | 392 (12.3%) | <0.001* |
| Bowel Injury | 0 (0.0%) | 7 (0.5%) | 0.001* | 32 (1.0%) | <0.001* |
| CBD Injury | 0 (0.0%) | 0 (0.0%) | 1.000 | 17 (0.5%) | <0.001* |
| Intra-op Cholangiogram | 62 (2.4%) | 178 (11.4%) | <0.001* | 497 (15.6%) | <0.001* |
| CBD Explored | 2 (0.1%) | 29 (1.9%) | <0.001* | 131 (4.1%) | <0.001* |

*p-Values compared the groups to those included in the paired analysis, and are from Chi^2^ tests for nominal variables, and Kendall’s tau for ordinal variables, unless stated otherwise.*

*^#^Reported as mean (SEM), with p-value from an independent samples t-test*

**Significant at p<0.05*
